# Injection of *Pseudomonas aeruginosa* Exo Toxins into Host Cells Can Be Modulated by Host Factors at the Level of Translocon Assembly and/or Activity

**DOI:** 10.1371/journal.pone.0030488

**Published:** 2012-01-27

**Authors:** Julien Verove, Cédric Bernarde, Yu-Sing Tammy Bohn, François Boulay, Marie-Josèphe Rabiet, Ina Attree, François Cretin

**Affiliations:** 1 INSERM, U1036, Biology of Cancer and Infection, Grenoble, France; 2 CNRS, ERL 5261, Bacterial Pathogenesis and Cellular Responses, Grenoble, France; 3 Université Joseph Fourier-Grenoble I, Grenoble, France; 4 CEA, DSV/iRTSV, Grenoble, France; Université de Genève, Switzerland

## Abstract

*Pseudomonas aeruginosa* type III secretion apparatus exports and translocates four exotoxins into the cytoplasm of the host cell. The translocation requires two hydrophobic bacterial proteins, PopB and PopD, that are found associated with host cell membranes following infection. In this work we examined the influence of host cell elements on exotoxin translocation efficiency. We developed a quantitative flow cytometry based assay of translocation that used protein fusions between either ExoS or ExoY and the ß-lactamase reporter enzyme. In parallel, association of translocon proteins with host plasma membranes was evaluated by immunodetection of PopB/D following sucrose gradient fractionation of membranes. A pro-myelocytic cell line (HL-60) and a pro-monocytic cell line (U937) were found resistant to toxin injection even though PopB/D associated with host cell plasma membranes. Differentiation of these cells to either macrophage- or neutrophil-like cell lines resulted in injection-sensitive phenotype without significantly changing the level of membrane-inserted translocon proteins. As previous *in vitro* studies have indicated that the lysis of liposomes by PopB and PopD requires both cholesterol and phosphatidyl-serine, we first examined the role of cholesterol in translocation efficiency. Treatment of sensitive HL-60 cells with methyl-ß-cyclodextrine, a cholesterol-depleting agent, resulted in a diminished injection of ExoS-Bla. Moreover, the PopB translocator was found in the membrane fraction, obtained from sucrose-gradient purifications, containing the lipid-raft marker flotillin. Examination of components of signalling pathways influencing the toxin injection was further assayed through a pharmacological approach. A systematic detection of translocon proteins within host membranes showed that, in addition to membrane composition, some general signalling pathways involved in actin polymerization may be critical for the formation of a functional pore. In conclusion, we provide new insights in regulation of translocation process and suggest possible cross-talks between eukaryotic cell and the pathogen at the level of exotoxin translocation.

## Introduction

*Pseudomonas aeruginosa* is a major Gram negative, opportunistic human pathogen associated with a variety of acute and chronic diseases. The bacterium can invade different tissues including respiratory and urinary tracts, damaged and burned skin, or injured cornea [1]. Patients with genetic disorder leading to cystic fibrosis are especially susceptible to chronic *P. aeruginosa* infection, which in turn contributes to morbidity and increased mortality. In the last decades, *P. aeruginosa* infections became a serious health problem as these bacteria are becoming multi-resistant to most existing antimicrobial treatments [2].

*Pseudomonas aeruginosa* is especially well equipped with numerous pathogenic mechanisms contributing to its virulence. Among six secretion systems found in Gram negative bacteria, *P. aeruginosa* possesses five of them, and some in several copies [3]. Type III secretion (T3S) machinery, conserved in a variety of Gram negative pathogens, is dedicated to export and translocation directly into eukaryotic cell cytoplasm of four exotoxins (ExoS, ExoT, ExoY and ExoU) [4]. These macromolecules have dramatic effects on signal transduction pathways and actin cytoskeleton, leading to cell dysfunction and, in some cases, cell mortality [5]. The passage of the exotoxins across the three membranes is performed by a complex nanostructure composed of at least twenty distinct proteins that associate in three sub-assemblies. The basal body, called the secreton, is spanning the two bacterial membranes by several superposed ring-like homo-oligomers [6]. Protruding outwards and in continuum with the basal body, the so-called T3S needle is composed of one protein, PscF in *P. aeruginosa*, that polymerizes on the bacterial surface to form a uniform 8-nm-wide channel supposed to be a conduit for exported substrates [7]. The first proteins proposed to be exported through the needle are PcrV, PopB and PopD, playing an essential role in toxin translocation across the plasma membrane of target cell [8], [9]. As a majority of T3S substrates, the three translocators are kept in bacterial cytoplasm in complexes with their cognate chaperons, PcrG for PcrV, and PcrH shared by PopB and PopD [10], [11], [12], [13]. PcrV belongs to a family of surface-exposed, soluble antigens, some of them shown to oligomerize on the secretion needle extremity [14], [15], [16], [17]. The essential role of PcrV in T3S function is clearly linked to insertion into the host membrane of two hydrophobic translocators, PopB and PopD [15], [18]. In all T3SS studied to date, the largest translocator (in *P. aeruginosa*, PopB) harbours two putative transmembrane domains, while the smallest protein (PopD) belongs to a subfamily of translocators possessing only one transmembrane domain [9], [11], [19]. After infection of erythrocytes, PopB and PopD remain associated with the plasma membrane, while the PcrV translocator stays bacteria-associated [15], [18]. Both translocators purified *in vitro*, bind to phosphatidylserine-charged liposomes and are capable of inducing the release of small-molecular weight substances [20]. Moreover, liposomes charged with cholesterol are particularly vulnerable and are disrupted in the presence of the translocator PopB [19] indicating the translocon preference for specific lipid environments. The translocator SipB of *Salmonella* has been shown to directly interact with cholesterol [21] and *Shigella* homologue IpaD binds to a lipid raft-associated eukaryotic protein CD44 [22]; in both cases cholesterol was shown to be required for efficient bacterial invasion of host cell [22], [23]. A regulation of the translocation process itself has been suggested for *P. aeruginosa* and *Yersinia* systems, where a translocated effector provides a feedback control by modulating an activity of yet-unknown host cell factor [8], [24]. Hence, the GTPase Activating Protein (GAP) domain of the effector YopE is essential for controlling *Yersinia* translocation efficiency probably by acting on actin cytoskeleton [25]. Recently, a unique *Yersinia* protein, YopK/YopQ, has been identified as playing an independent role in controlling YopB/D translocon function once being itself translocated into cells [26]. Furthermore, *P. aeruginosa* and *Yersinia* spp. T3SS show some host specificities both *in vivo* and *in vitro* [27], [28], [29], which might result from the differential expression of host components required for full T3S function. In this context HL-60 cells was the first cell line identified as non-permissive to ExoS intoxication [27]. In order to study the translocon function in *P. aeruginosa*-cellular infection model, we set up a quantitative effector translocation assay that relies on a ß-lactamase activity fused downstream of inactive ExoS or ExoY N-terminal domains. After infection, the activity of the reporter injected in cells was measured by flow-cytometry. The presence of Pop translocator proteins within host membranes was correlated with data on the translocation of reporter protein. This approach allowed us to demonstrate that insertion of translocators into membranes can occur without efficient effector translocation, as exemplified in T3S-resistant cells, HL-60 and U937. We showed that cholesterol-depleting agent methyl-ß-cyclodextrine, as well as several inhibitors of eukaryotic signal transduction pathways, inhibits the translocation of the reporters without affecting expression, export and association of Pop proteins with host cell membranes. These results further corroborate the idea that host-pathogen cross-talk is particularly important in T3S function and may influence the efficiency of effector injection.

## Results

We showed previously that the activity of T3S translocon of *P. aeruginosa* can be modulated *in vitro* by lipid content and pH [19], [20]. To analyse the translocon activity *in vivo*, we adapted a reporter system based on Bla/CCF2 enzyme/substrate combination [29] to *P. aeruginosa* T3SS. This system enables single-cell reproducible quantification of exotoxin translocation into host cells using a flow-cytometry based analysis.

### ExoS-Bla is an adequate reporter for measuring the T3S translocation efficiency

**Figure 1:**
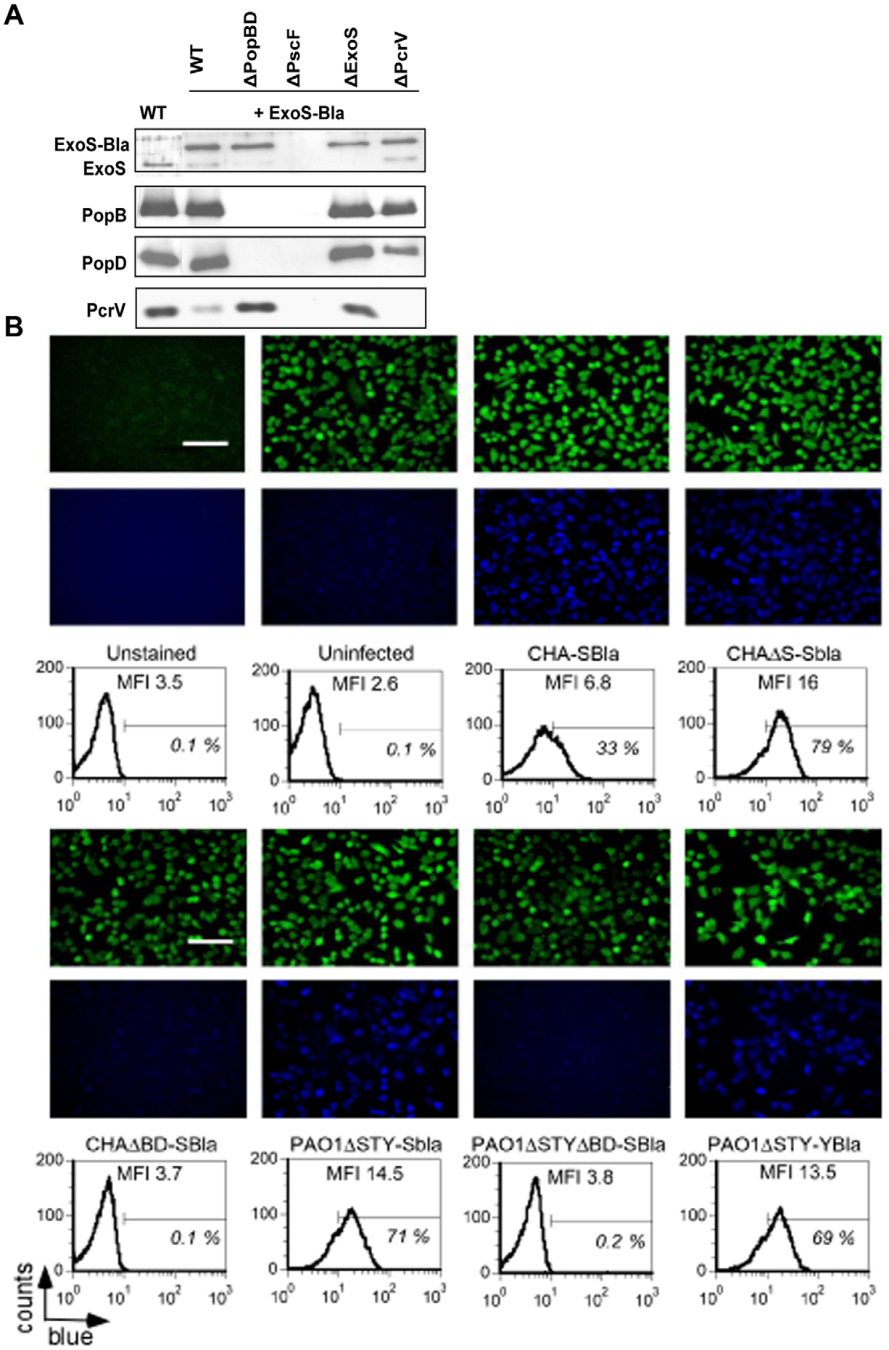
Characterization of ExoS-Bla and ExoY-Bla reporters. **A/** Secretion profiles of *P. aeruginosa* strains carrying the reporter fusion ExoS-Bla. Fifteen µL of culture supernatants of either wild-type CHA strain or mutant CHA strains expressing ExoS-Bla grown under T3SS-inducing conditions were analyzed by immunoblotting with antibodies directed against ExoS, PopB, PopD and PcrV. **B/** Co-cultures of A549 cells with *P. aeruginosa* strains for 3 h at MOI 10. Injection of either ExoS-Bla or ExoY-Bla fusions by strains CHA and PAO1ΔSTY was detected after incubating cells with β-lactamase substrate CCF2-AM either by fluorescence microscopy using a 20× objective (upper panel) or by flow cytometry (lower panel). The horizontal bar in flow-cytometry histograms indicates the gating used to determine the percentage of β-lactamase positive cells revealing ExoS-Bla injection. Mean Fluorescence Intensity is indicated in each panel. Scale bar, 100 µm.

The ExoS-Bla fusion was constructed so that its expression is directed by a cognate *exoS* promoter and the secretion and translocation of the fusion is achieved by the N-terminal ExoS sequences, in which the GTPase activating protein (GAP) activity was inactivated by the R146A mutation (see Materials and Methods). In-frame fusion with ß-lactamase was introduced in the different *P. aeruginosa* strains. The secretion of the fusion was evaluated by probing culture supernatants. As shown by immunoblotting using anti-ExoS antibodies (**Figure 1A**), the ExoS-Bla fusion was efficiently secreted from the wild-type *P. aeruginosa* strain CHA upon the induction of the system *in vitro* by Ca^2+^ depletion of the medium. The secretion of ExoS-Bla in the medium was strictly dependent on the functional T3SS needle composed by polymerized PscF. Translocon mutants, CHAΔPopB/D and CHAΔPcrV secreted ExoS-Bla but they were unable to inject the chimeric toxin in host cell cytoplasm (**Figure 1B**). To obtain maximal ExoS-Bla secretion, the endogenous *exoS* gene was deleted, so that no competition for the cognate chaperon and/or for other secreton components would influence full ExoS-Bla export. The translocation of ExoS-Bla was first tested on the epithelial cell line A549. After incubation of cells with different strains harbouring the fusion, the cells were incubated with ß-lactamase fluorescent substrate CCF2-AM and analysed by fluorescence microscopy. In parallel, the quantification was done by flow-cytometry based assay (**Figure 1B**). The CCF2 cleavage accomplished by a translocated fusion induces the disruption of FRET (Fluorescence Resonance Energy Transfer) and provokes a shift from green to blue fluorescence. While uninfected cells or cells infected with CHAΔPopB/D showed only a low mean fluorescence intensity (MFI) of about 3, the MFI reached a value of 16 when cells were co-incubated with the CHAΔS/ExoS-Bla strain (**Figure 1B**). Consistent with a competition between ExoS-Bla and endogenous ExoS observed *in vitro* (**Figure 1A**), the MFI observed with the strain CHA-SBla was 2.3 fold lower than that observed with CHAΔS-SBla (MFI 6.8 versus 16, respectively). The translocation efficiency was in the same range when using the CHAΔS-SBla strain (MFI 16) or the widely used PAO1 strain (MFI 14.5) deleted of all three exotoxins (PAO1ΔSTY, [31]) (**Figure 1B**). Consequently, all the following experiments were performed either with CHAΔS or PAO1ΔSTY strains. As observed in **Figure 1B**, the second reporter, ExoY-Bla, behaved as ExoS-Bla. Both reporters were translocated with the same efficiency in A549 cells infected with PAO1ΔSTY-YBla and PAO1ΔSTY-SBla (MFI 13.5 vs 14.5).

### Undifferentiated pro-myelocytic HL-60 and pro-monocytic U937 cell lines are resistant to T3S translocation

**Figure 2:**
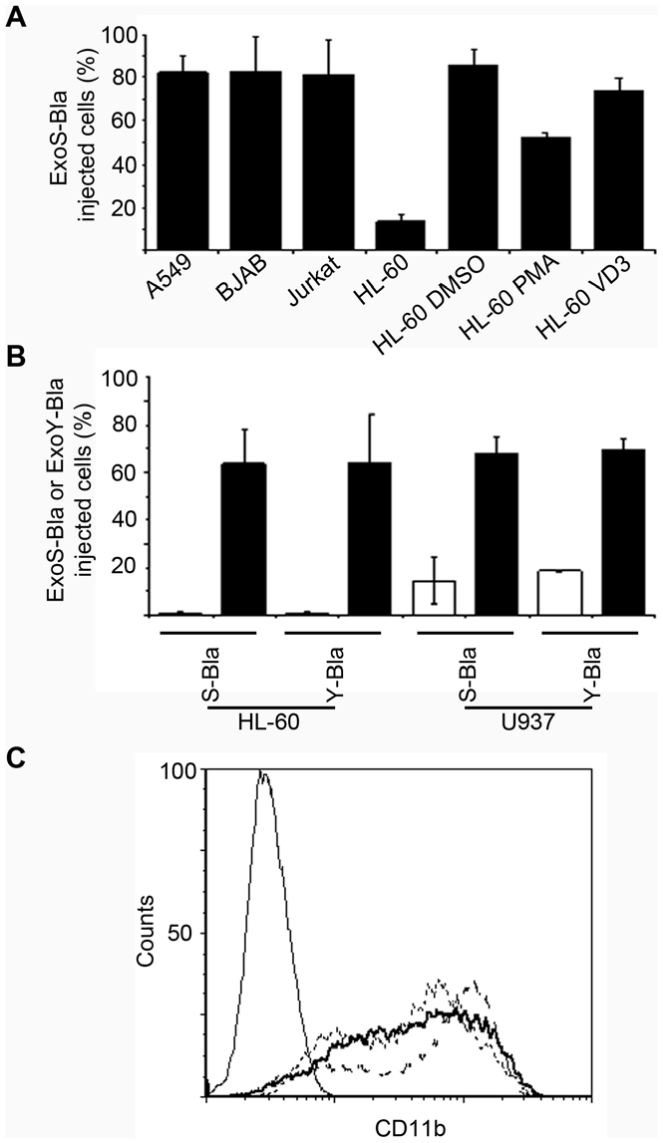
Efficiency of ExoS-Bla and ExoY-Bla translocation in different cell lines. **A/** A549 epithelial cells, BJAB, Jurkat, non-differentiated HL-60 cells (HL-60), or HL-60 differentiated into neutrophils, macrophages and monocytes by DMSO, PMA or Vitamin D3 (VD3), respectively, were infected at MOI of 10, for 3 h, with CHAΔS expressing ExoS-BlaR146A and analyzed by flow cytometry. **B/** HL-60 and U937 were differentiated in monocytes with VD3 (black bars) or not (white bars) and infected at MOI 10 with PAO1ΔSTY strains expressing either ExoS-BlaR146A or ExoY-Bla as described above. The error bars indicate standard deviation (n = 3). **C/** Non-differentiated HL-60 (thin lane) or HL-60 differentiated in neutrophils (dot line), monocytes (dash line) or macrophage (thick line) were labelled with a FITC conjugated antibody specific for CD11b and analysed by flow cytometry.

We then examined the translocation of ExoS-Bla in several eukaryotic cell types, including epithelial cell line A549, two lymphoid cell lines Jurkat and BJAB, as well as the pro-myelocytic HL-60 and the pro-monocytic U937 cell lines. As quantified by flow cytometry, epithelial and lymphoid cell lines were found sensitive to ExoS-Bla injection with about 80% of cells being injected at 3 h post-infection using a multiplicity of infection (MOI) of 10 (**Figure 2A**). In contrast, undifferentiated HL-60 and U937 cell lines were found refractory to ExoS-Bla and ExoY-Bla injection (**Figure 2B**). The inability to detect ExoS- or ExoY-Bla into resistant cells was not due to an artefact of inhibition or degradation of β-lactamase or to an inefficient loading of the CCF2 ß-lactamase substrate, since a HL-60 cell line that stably expressed ß-lactamase efficiently cleaved CCF2 (Data not shown). We then differentiated HL-60 cells into either neutrophil-like cells with dimethyl-sulfoxide (DMSO), macrophage-like cells with Phorbol Myristate Acetate (PMA) or monocyte-like cells with 1,2 3 dihydroxy-Vitamin D3 (VD3), as described in Materials and Methods. The differentiation state was assayed by following the expression of the macrophage/monocyte antigen, CD11b on cell surface by flow cytometry (**Figure 2C**). Interestingly, all three ways of differentiation provoked reversal from resistant to susceptible phenotype, albeit the injection of ExoS-Bla was significantly lower with macrophage-like cells (**Figure 2A**). These results corroborate previous observation showing that undifferentiated HL-60 cells are resistant to intoxication by ExoS [27] and identify a novel cell line (U937) showing a similar phenotype. The two undifferentiated cell lines were found to be resistant to injection of ExoY-Bla fusion, whereas differentiated cells were readily injected following infection (**Figure 2B**). Therefore, the resistance of pro-myelocytic and pro-monocytic cells is not limited to ExoS sequences, but could be linked to the general translocation process itself.

### Translocon insertion into HL-60 membranes is independent of cell differentiation

**Figure 3:**
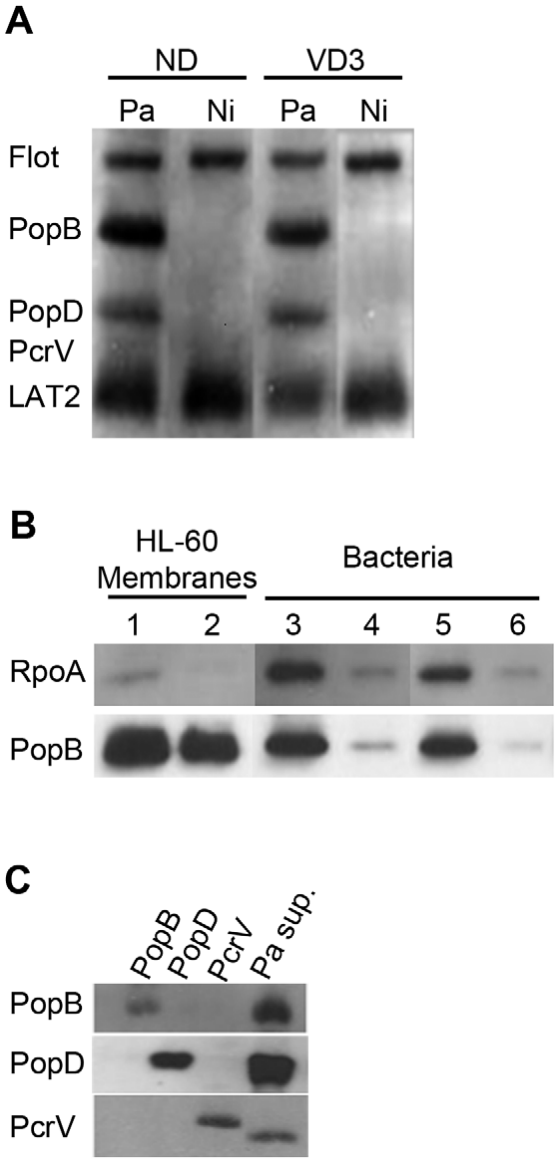
PopB/D proteins presence in the membrane of permissive and resistant cells is strictly due to translocon insertion. **A/** Non-differentiated HL-60 cells (ND) or VD3-differentiated HL-60 cells (VD3) were infected with CHAΔS-SBlaR146A for 3 h, at MOI 50, or not infected (Ni). After three washes, plasma membranes were purified by sucrose gradient centrifugation. The same amounts of plasma membrane proteins (7 µg) were separated by SDS-PAGE and revealed by immunoblotting with antibodies specific for PopB, PopD, PcrV, LAT2 or flotillin. **B/** After infection as above, non-differentiated and VD3-differentiated HL-60 cells were washed three times, treated to prepare plasma membrane fractions, and analysed by SDS-PAGE (lane 1 and 2, respectively) and immunoblotting for the presence of RpoA and PopB. Aliquots of supernatants corresponding to 2.10^6^ or 2.10^5^ bacteria incubated with undifferentiated HL-60 cells (lane 3 and 4) or with VD3-differentiated HL-60 cells (lane 5 and 6) were lysed and analysed as above. **C/** Calibration of antibodies used in panel A and B. 0.3 ng of recombinant proteins PopB, PopD and His-tagged PcrV and 20 µl of induced *P. aeruginosa* were loaded on SDS-PAGE, transferred and revealed by immunoblotting with antibodies specific for PopB, PopD or PcrV.

The absence of injection of ExoS-Bla and ExoY-Bla fusions within undifferentiated pro-myelocytic or pro-monocytic cell lines after infection could be due to the absence of translocon insertion. In *P. aeruginosa*, as well as in *Yersinia*, two hydrophobic translocators, PopB/YopB and PopD/YopD, insert into erythrocytes plasma membranes and could be detected by specific antibodies following cell fractionation by centrifugation on sucrose gradients [15], [18]. In these experiments, the hydrophilic translocators, PcrV/LcrV, are not associated with membranes and are used as a negative control for membrane purifications [15], [18]. To get further insights into the function of the translocon, we first examined the presence of Pop proteins in membranes derived from either injection resistant or injection permissive cells (i.e. undifferentiated HL-60 versus differentiated HL-60 cells, referred to as VD3-dHL-60 cells). To do so, infected host cell membranes were submitted to fractionation by centrifugation on sucrose gradients [30]. As expected, immunoblotting analysis of the different fractions revealed that PopB and PopD were readily inserted in the plasma membrane of permissive cells (**Figure 3A**). Both proteins were also found associated with membranes of non-permissive, undifferentiated HL-60 cells, suggesting that the defect of ExoS and ExoY translocation was not due to the absence of translocator proteins within the host cell membranes (**Figure 3A**). Similarly, PopB was recovered in the membranes from infected U937 cells independently of the differentiation status (data not shown). To ascertain that the detection of PopB/D was not resulting from a contamination of the membranes by bacteria, we first analysed samples of purified membranes for the presence of PcrV. As shown in **Figure 3A**, no PcrV protein could be detected by immunoblotting in the membrane prepared from infected HL-60 cells. This lack of detection is not due to the anti-PcrV antibody as evidenced by the detection of PcrV either in the supernatant of *P. aeruginosa* (**Figure 3C** **lane “** ***Pa*** **sup.”**) or as a His-tagged recombinant protein (**Figure 3C** **lane “PcrV”**). To further confirm that the presence of PopB/D in the membrane preparation was not due to residual bacteria, we used an antibody specific for bacterial RNA polymerase subunit A (RpoA). This antibody allowed the detection of as few as 2.10^5^ bacteria, i.e. 0.04% of the bacterial input used to infect cells. As illustrated in **Figure 3B**, only trace amounts of RpoA and PopB were detected in bacteria lysates corresponding to an input of 2.10^5^ bacteria (**lanes 4 and 6**). After infection of HL-60 cells, RpoA was hardly detectable in plasma membranes purified on sucrose gradient (**lanes 1 and 2**), whereas huge amounts of PopB were associated with membrane from undifferentiated HL-60 (**lane 1**) and VD3-dHL-60 cells (**lane 2**). This strongly suggests that the recovery of PopB/D results from the specific insertion of translocon in cell membranes and not from contaminating bacteria attached to infected cells. Altogether our results show that even though the injection of effectors occurs only in differentiated cells, the association of PopB/D translocators to membranes occurs equally well in both non-permissive and permissive cells. This suggests the implication of host components in translocon assembly and/or functionality.

### ExoS-Bla translocation occurs at the level of lipid rafts

**Figure 4:**
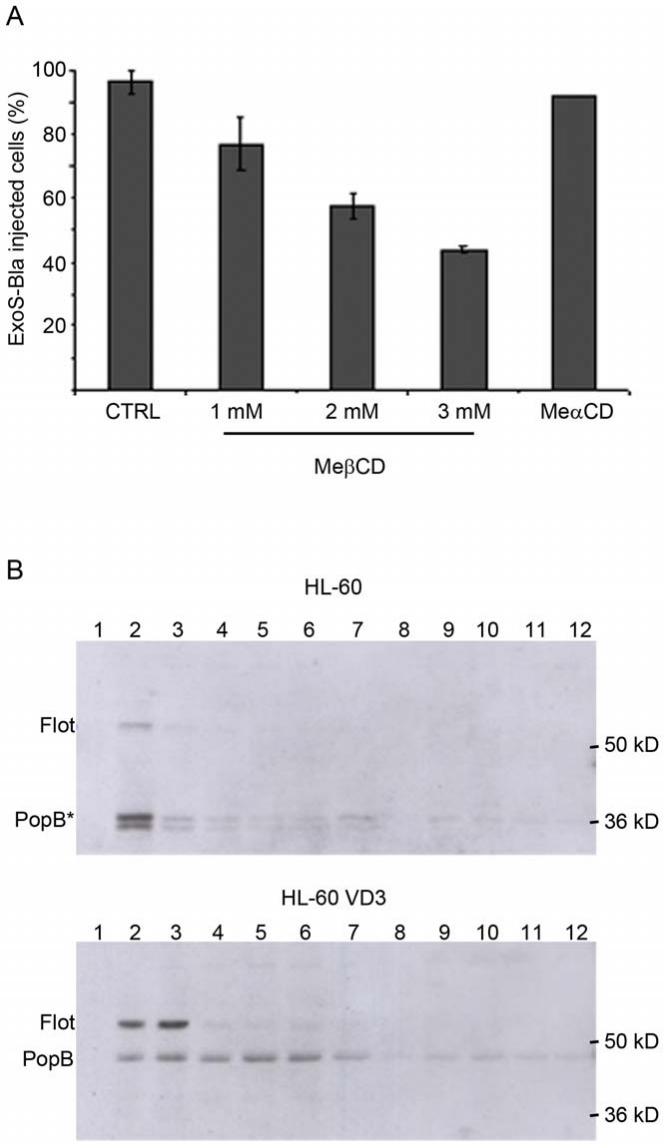
Cholesterol is essential for ExoS-Bla translocation in permissive HL-60 cells. **A/** VD3-differentiated HL-60 cells were grown as described in Materials and Methods and treated with 1 mM, 2 mM and 3 mM of Methyl-ß-cyclodextrine (MeßCD) for 2 h, before infection at MOI 10 with the CHAΔS-SBlaR146A strain. MeαCD an inactive isomer was used as control. The percentage of ExoS-Bla-injected cells was quantified by flow cytometry. The error bars indicate standard deviation (n = 3). **B/** Cholesterol-rich membranes (lipid rafts) were purified by flotation on sucrose step gradients after infection of cells at MOI 50 with the CHAΔS-SBla strain. For each fraction, the same amounts of proteins were separated by SDS-PAGE and analysed by immunodetection with antibodies specific for PopB or flotillin, a host marker for lipid rafts. * indicates a proteolysed form of PopB.

*In vitro*, *P. aeruginosa* translocators PopB and PopD efficiently disrupt artificial membranes (liposomes) only in presence of cholesterol [19], which is one of the host cell lipid components required for *Shigella* IpaB and *Salmonella* SipB translocator function [21], [23]. Moreover, cholesterol-rich membrane microdomains have been implicated in invasion of *P. aeruginosa* [31]. To investigate the role of cholesterol in exotoxin translocation, we treated permissive HL-60 cells with methyl-ß-cyclodextrine (MeßCD) and measured the level of ExoS-Bla injection. As shown in **Figure 4A**, the injection of ExoS-Bla was inhibited byMeßCD in a dose-dependent manner, reaching 50% of inhibition at 3 mM concentration. Higher concentrations of MeßCD induced cell mortality and were not taken into account. Of note, MeαCD, an inactive isomer of MeßDC, used at concentrations up to 20 mM, had no influence on ExoS-Bla injection. As cholesterol is the major building block of specialized glycolipoprotein micro-domains known as lipid rafts or detergent-resistant membranes (DRM), we specifically purified DRM from infected cells by ultracentrifugation on sucrose gradients following treatment with Triton X-100 at 4°C (see Materials and Methods), and the collected fractions were analysed for the presence of translocators. As shown on **Figure 4B**, in the case of infected VD3-dHL-60 cells, a portion of PopB was found preferentially associated with lipid rafts as it co-fractionated with flotillin, an eukaryotic lipid raft marker. Using the same protocol to purify lipid rafts from the injection resistant non-differentiated HL-60 cells, we systematically detected a truncated PopB protein (PopB*) that co-fractionated with flotillin. PopB was shortened by approximately 6 kDa, in spite of the addition of several cocktails of protease inhibitors. Thus, although PopB was recovered in both cases in the cholesterol rich membranes, it is differentially susceptible to proteolytic cleavage.

### Alteration of translocation efficiency by inhibitors of eukaryotic signalling pathways

**Figure 5:**
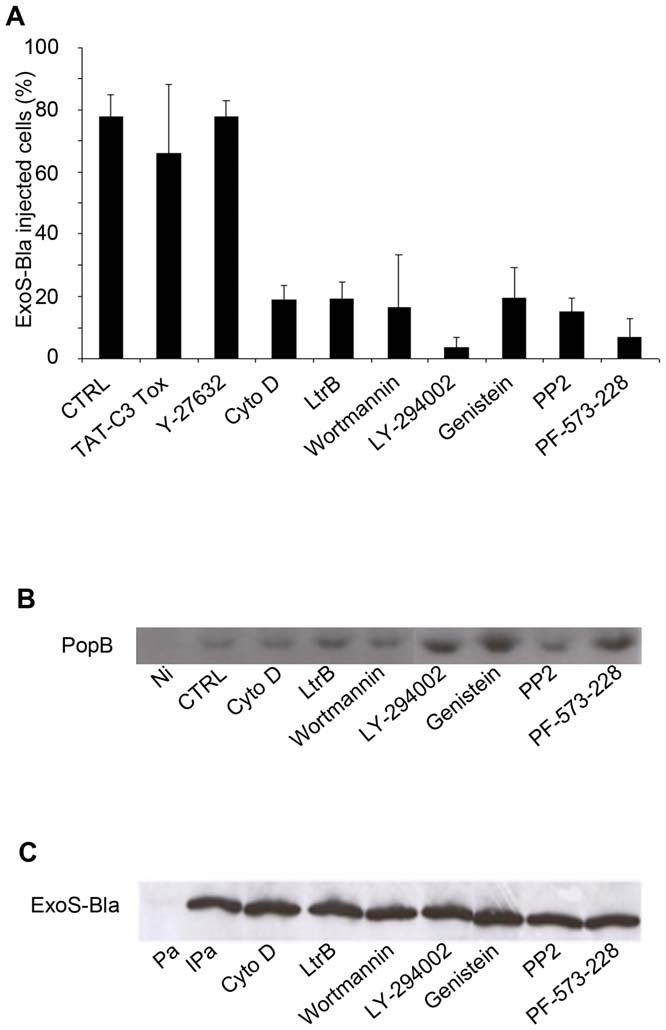
Injection of ExoS-Bla into differentiated HL-60 cells is sensitive to pharmacological agents. **A/** VD3-differentiated HL-60 cells were exposed to 10 µM of cytochalasin D (CytoD), 10 µM of latrunculin B (LtrB) or 50 nM of Wortmannin for 30 min prior and during infection, or to 2 µM of TAT-C3 toxin, 100 µM of LY-294002, 12 µM of genistein, 10 µM of PP2, and 10 µM of PF-573-228 for 120 min prior and during a 3 h infection period at MOI 10 with the PAO1ΔSTY-SBlaR146A strain. The percentage of injection-positive cells was evaluated by flow cytometry. **B/** Eukaryotic plasma membranes were purified by fractionation on sucrose gradient, after infection of cells pre-treated with inhibitors as above. Proteins were analysed by immunoblotting using anti-PopB. Ni : non-infected VD3-differentiated HL-60 cells, CTRL: infected VD3-differentiated HL-60 cells without inhibitor. **C/** The ability of PAO1ΔSTY-SBlaR146A strain to secrete ExoS-Bla *in vitro* was assayed for each inhibitor modifying injection by immunoblotting of total secreted ExoS-Bla protein using the anti-ß-lactamase antibody. Pa: supernatant of T3SS non-induced *P.aeruginosa*, IPa: supernatant of T3SS induced *P. aeruginosa* without inhibitor.

In order to decipher more in detail the eukaryotic elements and signalling pathways that influence the PopB/D translocon activity, a series of inhibitors were tested for their ability to impair the injection of ExoS-Bla within permissive VD3-dHL-60 cells by flow cytometry. None of the inhibitors had an effect on ß-lactamase activity itself, as verified by using a HL-60 cell line stably expressing ß-lactamase (not shown) or on T3SS dependent Exo-SBla secretion as shown in **Figure 5C**.

It is known that the level of cholesterol alters the viscoelastic properties of the plasma membrane and, thereby, regulates the actin-mediated deformation of the plasma membrane at the leading edge of moving cells [32]. We therefore examined whether the injection of ExoS-Bla required a functional actin network by treating cells during infection with two compounds affecting actin dynamics, cytochalasin D and latrunculin B [33]. At concentrations that did not alter cell viability, bacterial growth and T3SS dependent secretion *in vitro*, both molecules totally inhibited ExoS-Bla translocation (**Figure 5A**). As F-actin polymerization is under the control of the Rho family GTPases, we examined the translocation efficiency after treatment with inhibitors that directly target these GTPases or their effectors. In our hands, the translocation of ExoS-Bla was not impaired either by the cell permeable TAT-C3 toxin that targets RhoA, B and C or by the pharmacological compound Y-27632, an inhibitor of ROCK (Rho-controlled protein kinase). These two inhibitors had an inhibitory or a stimulatory action, respectively, on the ability of differentiated HL-60 cells to migrate through a 3 µm pore membrane to a source of chemoattractants (**Figure S1**). Thus, although they were unable to perturb ExoS-Bla injection, these two inhibitors of the Rho-dependent pathway were readily active.

Because the conversion of phosphatidylinositol (4,5)-bisphosphate into PtdIns(3,4,5)P_3_ by PI3-kinase promotes the actin-dependent uptake of *Yersinia pseudotuberculosis* [34], [35], *Listeria monocytogenes* [36], and *P. aeruginosa* [37], we tested two specific PI3-kinase inhibitors, wortmannin and LY-294002. As illustrated in **Figure 5A**, both compounds inhibited the translocation of ExoS-Bla. To investigate whether tyrosine kinases play any role in the functionality of the *P. aeruginosa* T3SS, several inhibitors were tested. ExoS-Bla injection into VD3-dHL-60 was abolished by genistein, a large spectrum tyrosine kinase inhibitor, by PP2, an inhibitor of the Src-kinase(s), and by PF-5732208, an inhibitor of focal adhesion tyrosine kinases, FAK and Pyk2 (**Figure 5A**). Most of the compounds showed a dose-dependent inhibition (**Figure S2**).

All the inhibitors that led to alteration in ExoS-Bla translocation were tested for their absence of effect on the association of PopB translocator with host cell membranes by applying sucrose-gradient fractionation. As shown in the **Figure 5B**, the association of PopB translocator with membranes of VD3-dHL-60 cells was not impeded when cells were treated with the different inhibitors. Thus, the insertion of the translocon proteins can be uncoupled from the translocation process *per se*.

**Figure 6:**
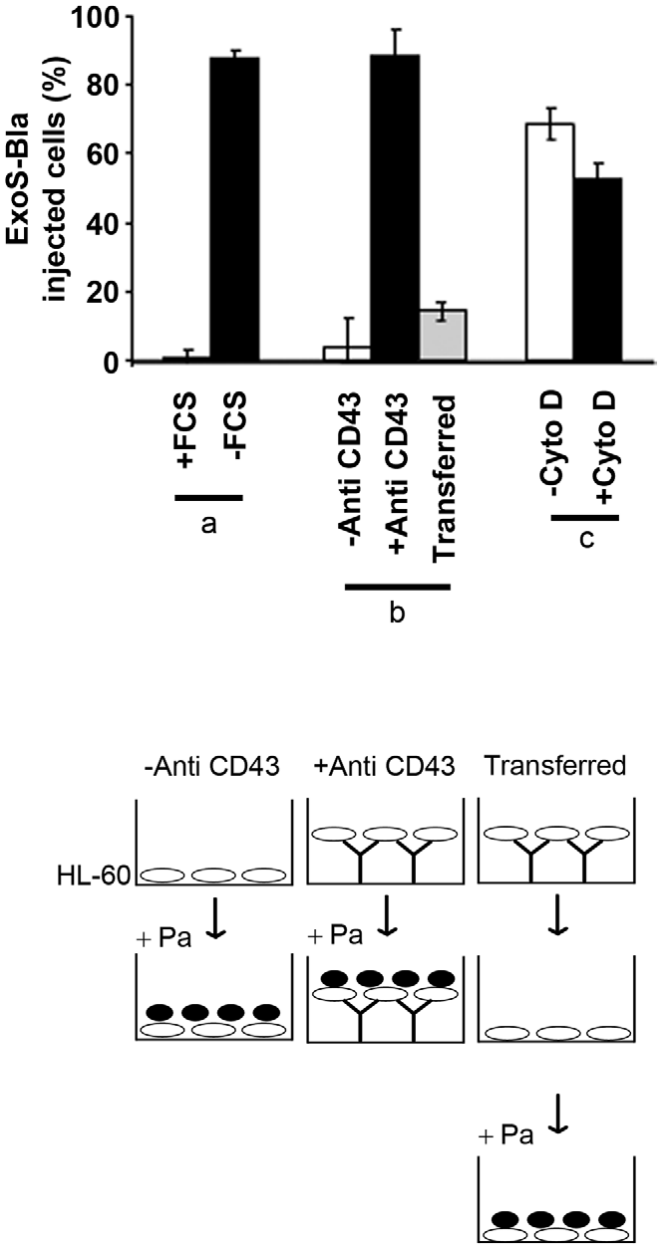
Switching T3SS resistant HL-60 cells to T3SS permissive cells by serum starvation or panning to anti-CD43. Non-differentiated HL-60 cells were treated as follows: maintained during 15 min in medium supplemented (+ FCS) or not (− FCS) (a), incubated during 5 min in either naive (− anti CD43) or coated wells with a monoclonal anti CD43 (+ anti CD43), first panned in a anti CD43-coated well during 5 min and then transferred to a naïve well, (transferred) (b) and cells were next incubated at MOI 10 with the CHAΔS-SblaR146A strain and analysed by flow cytometry. Non-differentiated HL-60 were incubated during 5 min in a well coated with anti CD43 in the absence (− CytoD) or the presence (+ CytoD) of 10 µM of cytochalasine D for 30 min prior and during infection at MOI 10 with the CHAΔS-SblaR146A strain (c). The error bars indicate standard deviation (n = 3). Insert: schematic drawing of the experiments presented in the histogram (part **b**).

A recent study by Bridge *et al*. suggests that the insertion of translocators into host cell plasma membrane requires cell adhesion and formation of leading edge [38]. Using two different approaches, we investigated whether adhesion of undifferentiated HL-60 cells could promote the translocation of ExoS-Bla. Cell adhesion was induced by transferring cells for a short period of time in culture medium devoid of foetal calf serum (FCS). Alternatively, cells were anchored in the presence of FCS on tissue culture plates previously coated with a monoclonal antibody specific for CD43, a cell-surface sialoglycoprotein highly expressed on HL-60 cells. As illustrated in **Figure 6**, both approaches yielded HL-60 cells that had rapidly switched from a translocation-resistant to a translocation-sensitive phenotype. In the presence of FCS, HL-60 cells originally resistant to ExoS-Bla translocation became sensitive when seeded in tissue culture plates pre-treated with anti CD43. Judging from the markedly reduced level of injected cells following the transfer of anti CD43-anchored cells in a well devoid of antibody (**Figure 6** **, part b**), it is clear that this acquired sensitivity was totally reversible and strictly dependent on cell immobilization on the tissue culture plate. To determine whether this acquired sensitivity to ExoS-Bla injection involved the same molecular mechanism as that used in VD3-dHL-60 cells, we tested the effects produced by cytochalasin D (**Figure 6** **, part c**). Surprisingly, cytochalasin D that did alter the injection of ExoS-Bla in VD3-dHL-60 cells, was unable to inhibit the injection of ExoS-Bla in cells immobilized through binding to anti CD43. Thus, the regulation of the functionality of T3S translocon appears to be divergent in VD3-dHL-60 cells and non-differentiated HL-60 cells that are turned on to be permissive to injection.

Altogether, our results show that the injection of exotoxins into the cytoplasm of VD3-dHL-60 cells is dependent on the integrity of the actin network and regulated by the level of cholesterol and the activation of host cell signalling pathways.

## Discussion

Within the sophisticated and complex T3S secretion machinery, the exact function and structure of translocator proteins is still a great mystery. It is believed that the T3S nano-machinery results from the complex assembly of proteins that form a continuum between the bacterium and the host cell membrane. Bacterial toxins are believed to reach the host cell cytoplasm after translocator proteins have formed a functional pore [39], [40], [41]. However, this microinjection model has recently been challenged as secreted effector proteins (extracellular) of *Yersinia pseudotuberculosis* were shown to be translocated in a T3SS-dependent mechanism [42]. In order to dissect translocon function *in vivo*, we adapted to the *P. aeruginosa* T3SS a simple translocation reporter system, which was first developed for *E. coli*, *L. pneumophilia*, *Y. pestis* and *P. aeruginosa* T3SS [26], [29], [43], [44], [45] and is based on the activity of ß-lactamase that cleaves a fluorescent substrate. In brief, the ß-lactamase was fused downstream of the N-terminal region of *P. aeruginosa* ExoS or ExoY toxins, which were both deprived of their enzymatic activities. The resulting chimeric proteins ExoS-Bla and ExoY-Bla were efficiently translocated into several cell lines such as the epithelial cell line A549, the B cell line BJAB, the T cell line Jurkat, and the differentiated promyelocytic HL-60 and promonocytic U937 cells as assessed by fluorescent microscopy and quantitative measurements using flow cytometry. When infection was performed with undifferentiated HL-60 or U937 cells, no injection of the reporter fusion could be observed.

The use of *P. aeruginosa* strains deprived of either ExoS (CHAΔS) or of the three ExoS, T and Y exoenzymes (PAO1ΔSTY) allowed a better intoxication of cells with ExoS-Bla or ExoY-Bla due to the absence of competition between the native exotoxins and the reporter fusion proteins for the access to the translocation machinery. Furthermore, the reporters ExoS-Bla or ExoY-Bla were engineered to remove the GAP and the adenylate cyclase activity, respectively, which excludes the possible implication of the reporter in the regulation of translocon activity and diversion of host cell signalling pathways.

To investigate whether host cell components play a role in the regulation of the general translocation process, we examined in parallel both the translocation efficiency and the presence of translocators PopB/D within plasma membranes of infected cells. PopB and PopD of *P. aeruginosa* are absolutely required for exotoxin injection into host cell cytoplasm without having any effects on effector secretion [18]. Both proteins possess predicted hydrophobic transmembrane helices and are prone to associate with lipids, both *in vitro* [19], [20] and *in vivo* [18]. Interestingly, in the present study, the observation that PopB and PopD are found associated with the membranes of injection-resistant and injection-sensitive cell lines suggests that the pore assembly and/or functionality may be regulated by host cell factors. The resistance of HL-60 cells to intoxication by exotoxins was recently correlated with the incapacity of bacterial cells to stably insert translocon proteins in the plasma membrane of non differentiated cells [38]. In our hands, immunoblotting analysis of plasma membrane purified on sucrose gradient did not reveal any degradation of either PopB or PopD whether cells were differentiated or not. However, PopB was found to be sensitive to proteolysis exclusively during the purification of specific membrane lipid rafts from the plasma membrane of non differentiated cells. The proteolysis of PopB may be due to an improper insertion in the plasma membrane of non differentiated HL-60 cells and, thereby, to an increased accessibility to proteases.

There is still little structural information on PopB/D proteins [9], probably due to their hydrophobic characteristics, molten globule conformation and/or oligomerization capacity [12], [19]. PopB shares secondary fold with its *Salmonella* orthologue SipB, for which exhaustive proteolysis experiments showed that it is deeply inserted into lipid bilayer, with the N-terminal region being exposed and cleaved off [46]. Indeed, *in vitro* studies have shown that PopB possesses a high pore forming activity whereas PopD activity is barely detectable and they do not associate with liposomes with the same efficiency [20].

Several parameters are likely to play a role in translocon assembly and/or functionality. It is known that an increase in cholesterol-to-phospholipid ratio enhances plasma membrane microviscosity in a large variety of cells [47]. Conversely, the chelation of cholesterol decreases the plasma membrane microviscosity and, thereby, redistributes proteins sequestered in specialized rafts to different regions of the plasma membrane. In the present study, we show that depletion of cholesterol from lipid rafts by methyl-ß-cyclodextrin abolished the translocation of the reporter ExoS-Bla in a dose-dependent manner. Co-localisation of PopB with the lipid raft marker flotillin in fractions of sucrose gradient is further supporting the role of lipid rafts in the translocation process. This is consistent with a previous study indicating that cholesterol is required for lysis of liposomes in presence of PopB [19]. Furthermore, specific membrane microdomains have been already involved in *Salmonella-* and *Shigella*-host cell interactions [21], [23]. Cholesterol accumulation results in increased membrane stiffness which could permit the formation of an actin network at the leading edge, an area recently proposed as the site of interaction between *P. aeruginosa* and differentiated HL-60 cells [38].

The actin network appears to play a major role in the translocation process in the VD3-HL-60 model as evidenced by the action of cytochalasin D and latrunculin B, two actin-depolymerizing agents which were found to markedly inhibit the injection of ExoS-Bla. It is worth noting that the effect of latrunculin B is much more pronounced in differentiated myeloid cells than in epithelial cells such as HT29 [38]. Unexpectedly, we were unable to observe any effect of either TAT-C3, or Y-27632, an inhibitor of ROCK, even though these inhibitors did enter into the cells and had a physiological effect as evidenced by their capacity to perturb the migration of VD3-HL-60 cells to a source of chemoattractants. Thus, the Rho-dependent pathway is unlikely to play a role in the formation of a functional pore. We could not conclude whether it is the integrity of the actin network rather than its dynamics that is the key parameter for translocon insertion and/or functionality.

In the VD3-dHL-60 model, the injection of ExoS-Bla is dramatically impaired when tyrosine kinases and PI3-kinase are pharmacologically inhibited. This observation is contrasting with the lack of effect reported by Bridge and coworkers with the epithelial HT29 model [38] or with lung-derived A549 epithelial cells (our unpublished data). In the context of VD3-dHL-60 cells, it is not known whether the functionality of the PopB/D pore depends on the basal activity of specific host cell signalling pathways or relies on the triggering of an “outside-in” signal underneath the site of bacterial attachment. In the case of *Yersinia enterocolitica*, Pyk2 and FAK, two tyrosine kinases localized in focal adhesion complexes, are required for invasin-mediated signalling through ß1-integrin [48]. The mechanism involved in the interaction between *P. aeruginosa* and VD3-dHL-60 is still unknown. Although a recent study suggests that ß-integrins are unlikely candidates since blocking antibodies directed to ß1 or ß2 integrins does not affect T3SS-mediated toxin injection [38], it is clear in our hands that the inhibition of FAK or Pyk2 markedly reduce the injection of the reporter.

Several recent reports on other pathogens harbouring T3SS suggest that the translocon proteins *per se* may be involved in host-pathogen cross-talk and signalling. The insertion of the translocation YopB/D pore into plasma membrane by *Y. enterocolitica* triggers maturation and release of pro-inflammatory cytokine IL-1ß, through the specific activation of caspase-1 [49]. A pathogenic strain of *Y. pseudotuberculosis*, depleted on all known T3SS-dependent effectors but possessing a functional translocon, activates cytosolic innate immune signalling through the activation of NF-κB and type I IFN-induced pathways [50]. *P. aeruginosa* devoid of effectors was shown to activate the NLRC4 inflammasome [51], [52], [53]. A direct role in translocation regulation was recently attributed to *Yersinia pestis* YopK/YopQ [26]. However, although a majority of components participating in assembly of T3S apparatus are highly conserved between *Yersinia* and *P. aeruginosa*, no homologue of YopK could be identified in *P. aeruginosa* genome, suggesting that there are alternative mechanisms to regulate translocation efficiency. In the case of SPI-2 T3SS of *Salmonella*, a pH modification was recently reported to play a key role in effector translocation and pore formation [54]. In a recent study, Cisz and coworkers [8] have shown that the functionality of T3SS required an unkown ExoS-inhibited host cell factor that may trigger either translocon insertion or stabilisation. We showed here that this may also occur without the participation of any (known) effector protein.

An important finding is that undifferentiated HL-60 cells that exhibit a resistant phenotype to T3SS-mediated toxin injection become rapidly susceptible to intoxication upon foetal calf serum depletion and adhesion to the culture plate or after panning in wells coated with anti-CD43. It is worth noting that the permissive HL-60 cells lose this acquired phenotype in the course of the infection step when they are transferred in naive wells devoid of anti-CD43. In this case the lack of toxin injection in undifferentiated HL-60 cells is not due to a defect in the expression of a specific gene. The mechanism underlying the T3SS-dependent toxin injection in anti-CD43 anchored cells may be different from that involved in VD3-dHL-60 cells. Indeed, while the injection process is fully inhibited in VD3-dHL-60 by agents that depolymerize the actin network, i.e. cytochalasin D and latrunculin B, the injection of ExoS-Bla in anti-CD43 treated cells was hardly inhibited by the same agents. A recent study by Bridge and coworkers establish a relationship between *P. aeruginosa* T3SS translocon function and the formation of a leading edge. Based on this model, we hypothesize that, as soon as anti-CD43 binds to CD43, non differentiated HL-60 cells reorganize their actin cytoskeleton, redistribute plasma membrane proteins into discrete domains and, subsequently, acquire a polarity. This notion is consistent with the fact that CD43 is a transmembrane glycoprotein known to concentrate at the uropod raft in activated polarized neutrophils [55] and T-cells [56]. The plasma membrane organization may orient the site of bacterial attachment and subsequently downstream signalling events that control pore formation.

Altogether, our results show that host cell components, such as cholesterol, actin cytoskeleton network and signal transduction pathways can modulate the injection of exotoxins into eukaryotic cytoplasm. The resistance to injection does not result from the absence of translocon insertion into host membranes. Therefore, the translocon activity and/or its proper assembly require multiple components of eukaryotic cell for efficient toxin translocation.

## Materials and Methods

### Bacterial strains, plasmid construction and analysis of proteins secretion

*P. aeruginosa* strains used in this study was cystic fibrosis clinical isolate CHA and its isogenic mutants CHAΔPscF [7] CHAΔPopBD [18]. For *ΔexoS* construction, the *exoS* gene and the flanking sequences were amplified by oligonucleotides ExoSss1 5′ggcccaggatcggcttgcaa and ExoSas2 5′gatccgctgccgagccaaga using DNA from the CHA strain as template. The PCR fragment of 1.8 kb was blunt-ligated into pEX100T [57]. The Gm cassette was extracted from pUCPGm [58] and inserted into pEX-ExoS. Inactivated copy of the *exoS* gene was introduced into CHA chromosome by allelic exchange as described [57]. CHAΔExoS mutant was checked by PCR and Western blotting. PAO1 depleted for the three exotoxins ExoS, ExoT and ExoY was kindly provided by Arne Rietsch [8], [59]. The *popBD* deletion was introduced into PAO1ÄSTY strain by double recombination procedure using the pJG5 plasmid as described [18]. The promoter region of *exoS* and the GAP-encoding part of the gene was PCR-amplified with primers pExoS1 5′ggatccacctgcaggctgagtac and exoSXba 5′ tctagacatcacttcggcgtcactgtg. The PCR product was cloned into pBluescript SK(+) and checked by sequencing. The fragment was excised by *Bam*HI-*Xba*I digestion and cloned into pUCP20 [58], giving pExoS_GAP_. The *bla* gene was amplified from pBR322 with primers βlacXba 5′ tctagacacccagaaacgctggtgaaag and βlacHind 5′aagctttttaccaatgcttaatcagtgaggcaccta, cloned as *Xba*I-*Hin*dIII into pExoS_GAP_ to give pExoS_GAP_Bla. The R146 mutation was introduced into pExoS_GAP_Bla by site-directed mutagenesis (Stratagene). The *exoY*-*bla* construction was created by PCR using pYEcos 5′ gaattccgccgcctcgccgagggtg and YXbaas 5′tctagaggcggtcatcgccagcccg which amplify 300 nucleotides upstream and 633 nt downstream of the ATG start codon of *exoY*, thus ending up just in front of the Asp_212_ codon known to be required for adenylate cyclase activity. The *exoY* PCR product was cloned in pBlunt-TOPO (Invitrogen) and sequenced, before cloning into pExoS-Bla in replacement of *exoS* sequence. The final plasmids were introduced into different *P. aeruginosa* strains by transformation [60]. For analysis of extracellular proteins, *P. aeruginosa* strains were grown to an optical density at 600 nm (OD_600_) of 1 in LB medium supplemented with 5 mM EGTA and 20 mM MgCl_2._

### Cell culture and incubation with *P. aeruginosa*

Promyelocytic cells HL-60, U937, Jurkat T cells, Bjab B cells and A549 epithelial cells (American Type Culture Collection, Manassas, VA) were cultured in RPMI 1640 with 2.0 mM L-Glutamine (Gibco) containing 10% FCS (Gibco), 100 U/ml penicillin and 100 µg/ml streptomycin (Gibco). Cells were maintained in a humidified atmosphere with 5% CO_2_ at 37°C. For infection, cells were washed once with complete RPMI medium without antibiotic and seeded in 24-well tissue culture plates (Falcon) at 10^6^ cells/ml in complete RPMI medium without antibiotic. *P. aeruginosa* strains were grown overnight at 37°C in LB containing 300 µg/ml of carbenicillin when needed, diluted the next day to an optical density at 600 nm (OD_600_) of 0.1 in the same medium and grown to OD_600_ of 1. The multiplicity of infection ranged from 10 to 50 and, infection was performed for 3 h in 5% CO_2_ at 37°C.

### Differentiation of myeloid cells

Cell differentiation into monocytes, macrophages and neutrophils was done by re-suspending the cells at 2.10^5^ cells/ml in complete RPMI culture medium containing 100 nM of 1,23 dihydroxy-Vitamin D3 (Sigma Chemical Co.) for 4 days, 5 ng/ml Phorbol Myristate Acetate (Sigma Chemical Co.) for 2 days and 1.25% DMSO (from Sigma Chemical Co.) for 5 days, respectively. Cell differentiation was assessed before each experiment by CD11b expression analysis using FITC conjugated antibody (IOTest®; Beckman Coulter) and flow cytometric analysis (FACScalibur).

### Panning conditions

24-well plate was incubated overnight in the presence of CD43 monoclonal antibody (clone L60, BD Pharmingen) at 1 µg/ml in PBS. After removing of the antibody solution and after three washes with PBS, the HL-60 cells were added in wells. The infection conditions were as described above.

### CCF2/AM loading and β-lactamase detection

After 3 h of infection, cells were incubated with freshly prepared 6×CCF2/AM solution (1 µM final concentration; Invitrogen). HL-60, Jurkat and Bjab cells were incubated for 30 min in the dark at room temperature. A549 cells were incubated 90 min in a 2 µM CCF2-AM in PBS. The percentage of cells that received reporter fusions was quantified by flow cytometry (FACS Moflo; Dako Cytomation). The results are expressed as percentage of cells that exhibit a blue fluorescence; uninfected cells incubated with CCF2 were used as negative control. Fluorescence micrographs were performed with a Cell-R inverted Olympus microscope using 20× objective.

### CD11b expression by FACS analysis

5.10^5^ non-differentiated or differentiated HL-60 cells were labelled with saturating amount of FITC conjugated monoclonal antibody specific for CD11b (clone Bear1). Labelled cells were acquired on FACSCalibur cytometer (Becton Dickinson).

### Treatment of cells by inhibitors

Prior to and during infection, cells were exposed to either 10 µM of cytochalasin D, 10 µM of latrunculin B or 50 nM of Wortmannin (Sigma Chemical Co.) for 30 min, at 37°C. Cells were also exposed to either 100 µM of ROCK inhibitor Y-27632, 2.5 nM of Toxin B from *Clostridium difficile* (Sigma Chemical Co), 2 µM of TAT-C3 toxin from *Clostridium botulinum* (Cytoskeleton), 10 µM of PP2, 10 µM of PF-573-228 (Calbiochem), 100 µM of LY-294002 (Sigma Chemical Co) or 12 µM of Genistein (Sigma Chemical Co) for 120 min prior to and during infection in a humidified atmosphere with 5% CO_2_ at 37°C. For methyl-β-cyclodextrine (MeßCD) treatment, HL-60 cells were grown in RPMI supplemented with 1% Nutridoma-SP (Roche) to diminish cholesterol concentration of the medium. Then, cells were treated with 1, 2 or 3 mM MeßCD (Sigma) for 2 h before infection at 37°C in RPMI supplemented with 1% Nutridoma-SP and for 3 h during infection. The cell viability was controlled by Trypan Blue exclusion.

### Preparation of plasma membranes by sucrose discontinuous gradient centrifugations

Eukaryotic plasma membranes were purified after infection using a previously described procedure [30]. Briefly, 60.10^6^ HL60 cells at a density of 10^6^ cells/ml were infected during 3 h at MOI 50 in RPMI supplemented with 10% FCS. Cells were then washed 3 times in cold PBS, and finally resuspended in 900 µl of cold buffer A containing 3 mM imidazole, 250 mM sucrose, 0.5 mM EDTA and proteases inhibitors cocktail (PIC, final concentration of 1/100 v/v, P8340 SIGMA), pH 7.4. All steps were performed at 4°C. Cells were lysed by 40 passages through a 25-gauge needle. Lysates were centrifuged 15 min at 850 g, and 750 µl of supernatant were homogenized with 1.5 ml of buffer containing 3 mM imidazole, 63% sucrose and PIC, and placed into ultra-clear centrifuge tubes (Beckman Coulter). One ml of buffer containing 3 mM imidazole, 40% sucrose and PIC was gently added and subsequently recovered with 1 ml buffer A. Samples were centrifuged 16 h at 15000 g in a SW50.1 rotor from Beckman Coulter. Then, the top 1.75 ml were gently harvested, homogenized with 875 µl cold PBS containing PIC, and centrifuged 30 min at 166000 g (rotor TLA110, Beckman Coulter). Pellet was harvested in 100 µl cold PBS containing PIC, and protein concentration determined with Bradford Reagent (Sigma-Aldrich). Thus, the same quantities of proteins for each sample were processed for immunoblotting.

### Detergent resistant membrane (DRM) extraction

After infection, all steps were conducted at 4°C. After infection of 1.8.10^8^ HL-60 cells (MOI 50 for 3 h and a density of 10^6^ cells/ml), infected cells were washed once with cold RPMI and lysed in 1.5 ml MBS (25 mM MES, 150 mM NaCl, pH 6.5) containing 1% Triton X-100, 1/100 PIC, and 1 mM benzamidine (B6506, Sigma-Aldrich). Detergent treated membranes were centrifuged for 10 min at 3220 g and supernatant was homogenized with 2 ml of MBS containing 85% sucrose into an ultracentrifuge tube, and further covered with 6.3 ml of MBS containing 35% sucrose and 2 ml of MBS containing 5% sucrose. Each sucrose solution contained PIC and 1 mM benzamidine. Samples were centrifuged at 260000 g for 18 h at 4°C in a Beckman Coulter SW41 rotor. DRM-containing fractions were harvested at 35% sucrose/5% sucrose interface, diluted with 1.4 ml MBS containing 1/100 PIC and 1 mM benzamidine, and centrifuged at 376000 g for 30 min. Pellet was recovered with 100 µl MBS containing PIC and 1 mM benzamidine, and protein content was determined with Bradford Reagent.

### Immunoblotting analysis

Following sucrose gradients, equal quantities of protein (determined by Bradford method), were subjected to 12% SDS-PAGE, and further transferred to nitrocellulose membrane. For antibody calibrations, purified proteins 6His-PcrV, PopB and PopD [16], [19] were diluted in PBS buffer and 0.3 ng of each was loaded on the same gel. The antibodies were previously described [18]. They were affinity-purified and used at the following dilutions: anti-PopB (1∶5000), anti-PopD (1∶3000) anti-PcrV (1∶3000). Mouse monoclonal anti-RpoA was purchased from Neoclone Biotechnology and used at 1∶1000 dilution. Rabbit polyclonal anti-flotillin-2 (Santa Cruz Biotechnology) and mouse monoclonal anti-NTAL (Santa Cruz Biotechnology) were used at 1∶200 dilution. ECL™ anti-Rabbit or anti-mouse IgG HRP conjugated secondary antibodies (GE Healthcare) were used at 1∶5000 dilutions. The membrane was developed by ECL kit (GE Healthcare).

### Chemotaxis assay

The chemotaxis assay has been previously described [61]. In brief, VD3-differentiated HL-60 cells were centrifuged, resuspended in fresh complete RPMI 1640 medium at the density of 10^6^ cells/ml and incubated overnight with 5 nM of 1,1′-dioctadecyl-3,3,3′,3′-tetramethylindolcarbocyanine perchlorate (Molecular probes, Eugene, OR). Cells were then centrifuged and washed once with complete medium and twice with the chemotaxis buffer (RPMI 1640 supplemented with 5% heat inactivated foetal calf serum). Cells at a density of 10^6^ cells/ml were preincubated, for 2 h at 37°C, in the chemotaxis buffer in the presence of either the vehicle (50% glycerol in phosphate buffered saline) or the inhibitors, including TAT-C3 (0,33 µM), a cell permeable toxin targeting RhoA, B, and C, or 100 µM of the pharmacological compound Y27632, an inhibitor of ROCK. One hundred µl of cells (10^5^ cells) treated or not with inhibitors were loaded into the upper chamber of FluoroBlok inserts of 3 µm pore size (Becton Dickinson, Le Pont-de-Claix, France). Five hundred µl of a 10-fold dilution of *P. aeruginosa* culture supernatant were placed in the lower chambers and the FluoroBlok inserts were incubated at 37°C, for 4 h. Migrating cells dropped from the filters to the bottom of the lower chamber were counted in five fields (magnification×100) using an inverted fluorescence LEICA DMIER2 microscope Three independent set of experiments were performed and results are presented as the percentage of cells migrating through the filter in control chambers, i.e. without inhibitors.

## Supporting Information

Figure S1 **Effects of Rho GTPase and Rho kinase inhibitors on the migration of VD3-differentiated HL-60 cells.** To determine whether TAT-C3 (0,33 µM), or the ROCK inhibitor Y27632 (100 µM) were readily active and had a physiological effect on VD3-differentiated HL-60 cells, we examined whether they had the capacity to alter the migration of VD3-differentiated HL-60 cells through a 3 µm pore membrane to a source of chemoattractants, namely the supernatant of cultures of *P. aeruginosa* growing in the exponential phase (see Materials and Methods). In each set of experiment, we counted the number of cells present in the lower chamber either in the absence of inhibitor (control) or in the presence of inhibitors in five microscopic fields chosen at random (magnification×100). Results are presented as migrating cells expressed in percent of cells migrating in the control.
(TIF)

Figure S2 **Dose effect of pharmacological agents.** HL-60 VD3 were exposed to different concentrations of cytochalasin D (cytoD), latrunculin B (LtrB), wortmannin for 30 min prior and during infection or to different concentrations of LY-294002, Genistein, PP2 or PF-573-228 for 120 min prior and during infection. HL-60 VD3 were infected at MOI of 10, for 3 h, with PAO1F Δ3STY ExoSBlaR146A strain and then analysed by flow cytometry.
(TIF)
