# Supplementary figures and images for: Injection of Pseudomonas aeruginosa Exo Toxins into Host Cells Can Be Modulated by Host Factors at the Level of Translocon Assembly and/or Activity
Source: PLoS One. 2012 Jan 27;7(1):e30488. doi: 10.1371/journal.pone.0030488 (PMC3267729; doi:10.1371/journal.pone.0030488)

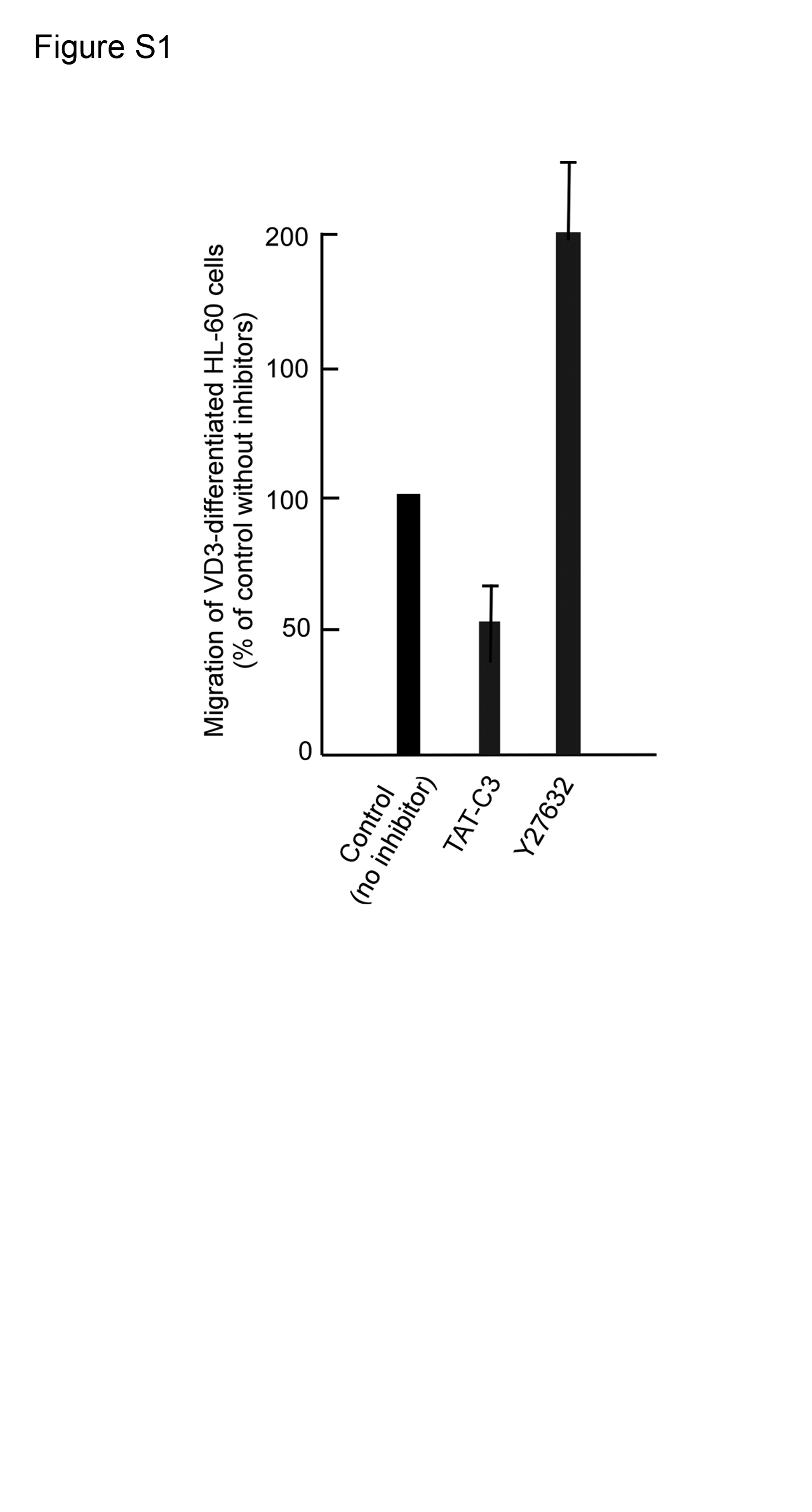

Supplement: Figure S1 — Effects of Rho GTPase and Rho kinase inhibitors on the migration of VD3-differentiated HL-60 cells. To determine whether TAT-C3 (0,33 µM), or the ROCK inhibitor Y27632 (100 µM) were readily active and had a physiological effect on VD3-differentiated HL-60 cells, we examined whether they had the capacity to alter the migration of VD3-differentiated HL-60 cells through a 3 µm pore membrane to a source of chemoattractants, namely the supernatant of cultures of P. aeruginosa growing in the exponential phase (see Materials and Methods). In each set of experiment, we counted the number of cells present in the lower chamber either in the absence of inhibitor (control) or in the presence of inhibitors in five microscopic fields chosen at random (magnification×100). Results are presented as migrating cells expressed in percent of cells migrating in the control. (TIF) [file pone.0030488.s001.tif]

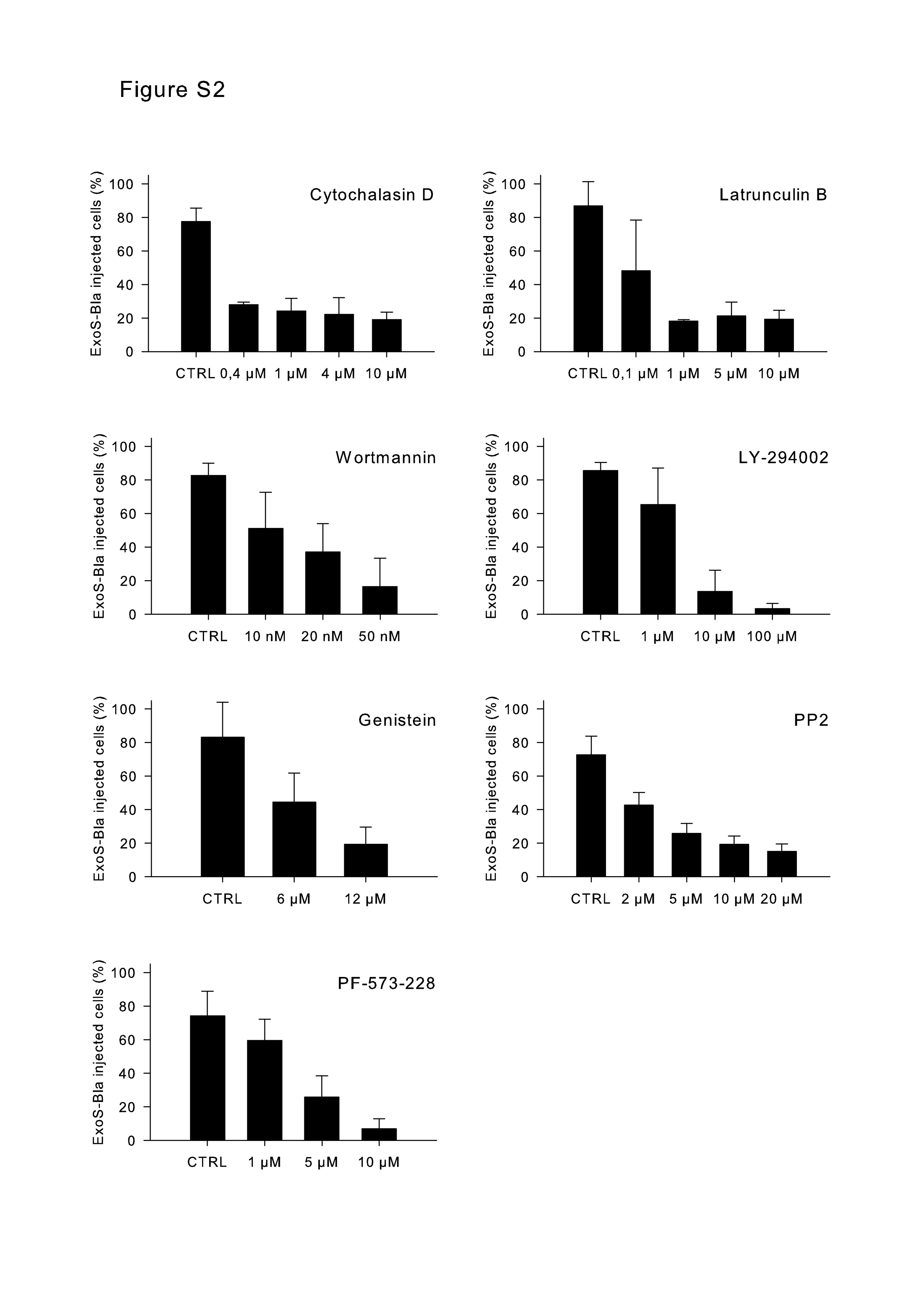

Supplement: Figure S2 — Dose effect of pharmacological agents. HL-60 VD3 were exposed to different concentrations of cytochalasin D (cytoD), latrunculin B (LtrB), wortmannin for 30 min prior and during infection or to different concentrations of LY-294002, Genistein, PP2 or PF-573-228 for 120 min prior and during infection. HL-60 VD3 were infected at MOI of 10, for 3 h, with PAO1F Δ3STY ExoSBlaR146A strain and then analysed by flow cytometry. (TIF) [file pone.0030488.s002.tif]
